# Cohesin protein Smc3 influences kinocilial structure and function

**DOI:** 10.1242/bio.062029

**Published:** 2025-12-11

**Authors:** Fiona M. Mensching, Niusha Banoukh, M. Kathryn Iovine

**Affiliations:** ^1^ Department of Biological Sciences, Lehigh University, 111 Research Drive, Bethlehem, Pennsylvania 18015, USA; ^2^ Department of Neurology, Boston Children's Hospital, 300 Longwood Avenue, Boston, Massachusetts 02115, USA

**Keywords:** Smc3, Hair cells, Zebrafish, Otoliths, Kinocilia, Cohesins

## Abstract

Cohesinopathies and ciliopathies are congenital disorders affecting overlapping body systems. The extent to which these syndromes may be linked remains largely untested. Recently, reduced expression of a cohesin core subunit, Smc3, was found to result in abnormal otolith development in zebrafish embryos. This finding suggests that Smc3 may contribute to kinociliary development and function, which would represent a novel role for Smc3. Using hair cells found in neuromasts of the posterior lateral line, we found that Smc3 knockdown resulted in reduced kinociliary length. To address the role of Smc3 in kinocilial function, we monitored neomycin resistance of neuromasts (associated with several cilial gene mutants) and FM1-43X uptake in hair cells (associated with mechanotransduction). We found that Smc3 knockdown indeed led to neomycin resistance of the posterior lateral line neuromasts, suggesting impaired kinocilium function. However, neuromast hair cells did not have defects in FM1-43X uptake. We further demonstrated that hair cell number is reduced within neuromasts. This study suggests a significant influence of cohesin subunit Smc3 in ciliary structure and function and provides a preliminary link between cohesinopathy and ciliopathy etiologies.

## Teaser

**Summary:** This study finds that cohesin complex subunit Smc3 influences the development of zebrafish hair cell kinocilia, suggesting a potential link between cohesinopathy and ciliopathy etiologies.

## INTRODUCTION

The zebrafish cohesin protein complex is composed of core subunits Smc1, Smc3, and Rad21 that form a ring-like structure upon which a variety of auxiliary proteins (Wapl, Pds5, and Stag1-3) are recruited (Islam et al., 2021; Rankin, 2015). Cohesins are ATPase complexes that impact DNA in two fundamental ways. Early studies revealed that cohesins tether together sister chromatids – a process critical for high fidelity chromosome segregation (Skibbens, 2000). Later studies reported that cohesins also extrude DNA from within a single chromosome to form near mega-base sized loops (Hadjur et al., 2009; Mishiro et al., 2009; Wendt et al., 2008). These loops, or topologically associated domains (TADs), are posited to demarcate transcriptional states critical for proper development (Rajderkar et al., 2023). Cohesin gene mutations thus result in maladies such as Roberts syndrome (RBS) and Cornelia de Lange syndrome (CdLS), which are collectively known as cohesinopathies (Piché et al., 2019; Banerji et al., 2017). Cohesin gene mutations, or alterations in cohesin levels, however, are also associated with numerous forms of cancer (Duesberg et al., 2008; Holland and Cleveland, 2012; Santaguida and Amon, 2015; Vasudevan et al., 2021).

Cohesinopathies, such as RBS and CdLS, exhibit wide-ranging symptoms that extend across multiple organ systems. Prevalent phenotypes include abnormal limb development, craniofacial deformities, cardiac defects, and hearing loss (Piché et al., 2019). In addition to hearing loss, patients with CdLS have been noted to have significant, externally visible ear deformities, though defects to otoconia have not been reported (Ansari et al., 2024). Zebrafish larvae with deficiencies in Smc3, the acetyltransferase (Esco2) that regulates cohesin dynamics, or in auxiliary proteins Nipbla and Nipblb, exhibit various phenotypes including decreased eye size, body length, curved-down tail, and otolith abnormalities (Sanchez et al., 2022, 2025). Knockdown of the cohesion regulator Nipbl produces various heart and gut defects, disruption of endodermal gene expression, and left-right patterning defects (Muto et al., 2011).

Interestingly, curved-down tail and laterality phenotypes are both common among cilia gene mutants in zebrafish (Brand et al., 1996; Haffter et al., 1996). Cilia are microtubule-based organelles that serve as signaling antennas and are particularly important during development (Whitfield, 2020; Klena and Pigino, 2022). Microtubules comprise the axoneme of this antenna. In addition, microtubules serve as railroad-like tracks for the bidirectional transport of cargo in and out of the cilium, a process known as intraflagellar transport (IFT) (Taschner and Lorentzen, 2016; Klena and Pigino, 2022). Surrounding the axoneme is the ciliary membrane, which is continuous with the cellular membrane (though possesses a distinct protein/lipid composition; Klena and Pigino, 2022). Governing the separation of the organelle from the rest of the cell is the transition zone, marked by transition fibers and Y-links that connect the basal body and microtubule stalks to the ciliary membrane, respectively (Garcia-Gonzalo and Reiter, 2017).

Prior evidence implicates cohesins in cilia structure/function. For example, both SMC1 and SMC3 were found to co-immunoprecipitate with a cilia membrane protein in MDCK cells (Khanna et al., 2005). Separately, SMC1 was found to associate with the basal body (i.e. the intracellular anchor for cilia) in four different mammalian cell lines (Guan et al., 2008). Shugoshin (a cohesin-associated factor), was found to localize to the basal body in *C. elegans* (Reed et al., 2023). Furthermore, mutations in a gene coding for a cohesin-associated protein causes ciliopathy-like symptoms in two unrelated children (Scheuerle et al., 2023). The NudC-like protein 2 (NUDCD2) binds cohesins (Yang et al., 2019), localizes to centrosomes (Yang et al., 2010, 2019), and regulates centrosome duplication (Li et al., 2019). Together, these studies suggest that cohesin subunits may associate with or near cilia, but the extent to which cohesin proteins impact ciliary function has not been tested.

Otoliths are mineralized structures located in the vertebrate ear used to sense movement. In zebrafish, otolith formation relies on kinocilia, which are specialized primary cilia found along with actin-based stereocilia in sensory hair cells (Whitfield, 2020; O'Donnell and Zheng, 2022). Hair cells occur in both the otic vesicle and (in zebrafish) in sensory neuromasts located along the lateral line (Wang and Zhou, 2021). For example, the posterior lateral line is comprised of 12 neuromasts spaced along the lateral aspect of the zebrafish body and tail (Dijkgraaf, 1963; Ghysen and Dambly-Chaudière, 2007; Thomas et al., 2014). Each neuromast contains clusters of hair cells that serve mechanosensory functions, such as detecting changes in water flow (Wang and Zhou, 2021; Thomas et al., 2014). Previously, we reported that knockdown of Smc3 or Esco2 resulted in abnormal otolith number, size, and positioning, in zebrafish otic vesicles (Sanchez et al., 2022). While *smc3* is expected to be expressed ubiquitously, in addition, recent single cell RNA-sequencing (scRNA-seq) datasets demonstrate that *smc3* is expressed in hair cells of the otic vesicle and lateral line as early as 24 hours post fertilization (hpf) (Sur et al., 2023; Farnsworth et al., 2020). Therefore, due to the observed otolith phenotypes in cohesin depleted embryos, in combination with evidence that *smc3* is expressed in hair cells, here we test if Smc3 contributes to kinocilia structure and/or function.

## RESULTS

### Smc3 function contributes to kinocilia length and hair cell number in neuromasts

**Fig. 1.**
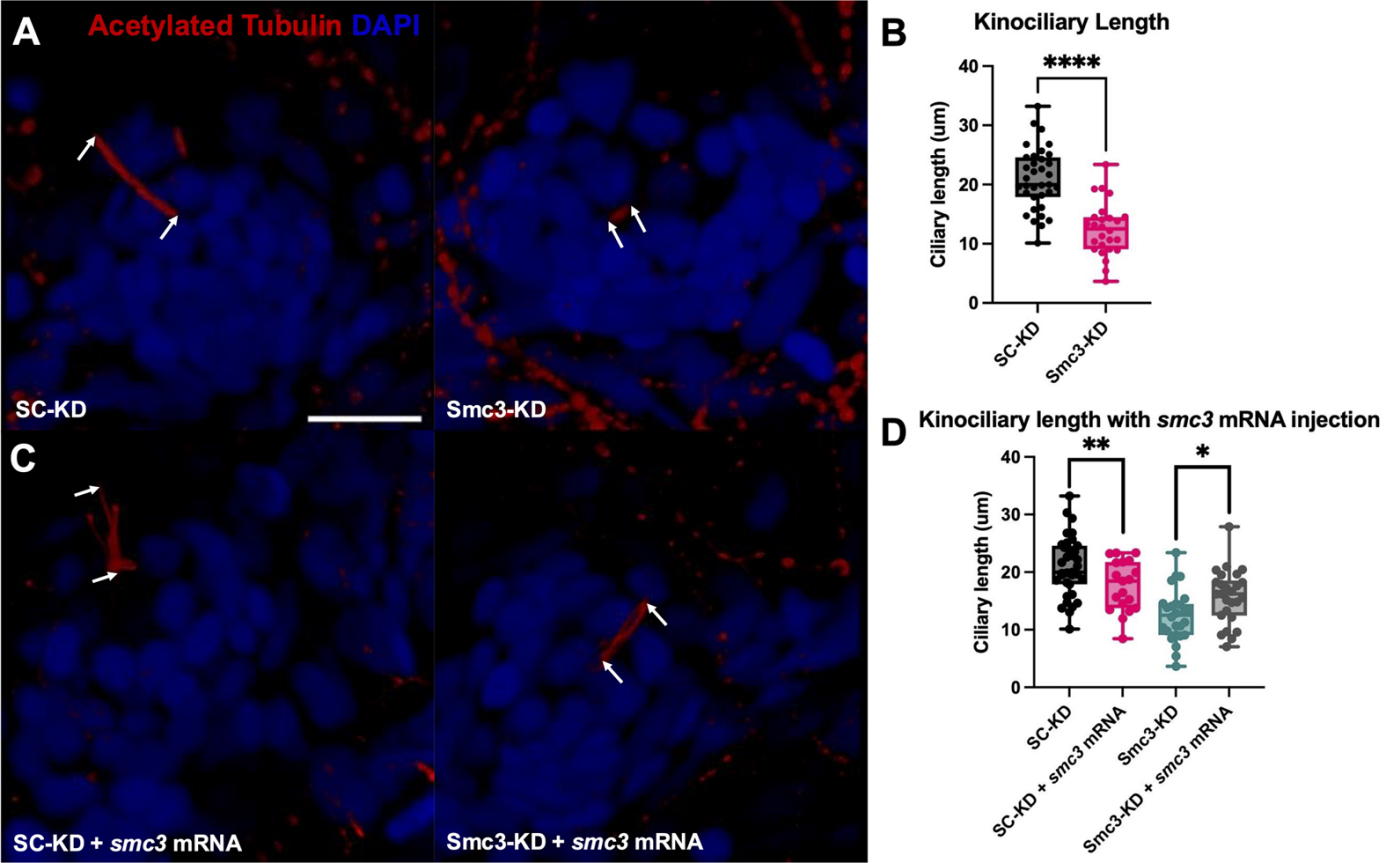
**Smc3-KD leads to decreased kinociliary axoneme length in the first neuromast along the posterior lateral line, which is rescued by *smc3* mRNA overexpression.** (A) Representative images of the first posterior lateral line kinocilium in SC-KD (left), Smc3-KD (right). Larvae were fixed at 3 dpf. Acetylated tubulin is labeled in red, and DAPI is in blue. Arrows identify the base and tip of each measured kinocilium. Scale bar: 10.4 µm in the x and y dimensions. (B) Quantification of the decrease in axoneme length in Smc3-KD (Student's *t*-test, ****P<0.0001, SC-KD *n*=35, Smc3-KD=26). (C) Representative images of the first posterior lateral line kinocilium in SC-KD+*smc3* mRNA (left), and Smc3-KD+*smc3* mRNA (right). Scale bar: 10.4 µm. (D) *smc3* mRNA co-injection in Smc3-KD rescues axoneme length as compared Smc3-KD alone (Student's *t*-test, **P=0.0123, Smc3-KD+*smc3* mRNA *n*=26, Smc3-KD *n*=26) *smc3* mRNA co-injection in SC-KD decreases axoneme length as compared SC-KD alone (Student's *t*-test, *P=0.0228, SC-KD+*smc3* mRNA *n*=19, SC-KD *n*=35).

To investigate whether Smc3 is implicated in kinociliary development, we evaluated the impact of reduced Smc3 expression on the organelle. Zygotes were injected with morpholino oligomers (MOs) targeting Smc3 (validated in Ghiselli, 2006 and used Sanchez et al., 2022), or a standard control (SC) MO. Phenotypes were evaluated at 3 days post fertilization (dpf) in the hair cells in the lateral line, which is often used as a proxy for hair cells in the otic vesicles due to their accessibility (Brignull et al., 2009). Importantly, Smc3 protein levels were reduced by 87% in Smc3-KD larvae at 3 dpf (Fig. S1). For consistency across larvae, we focused on the length of the longest of multiple observed kinocilial axonemes extending from the first posterior lateral line neuromast from one side of each embryo (see the Materials and Methods and Fig. S2 for images showing the presence of multiple kinocilial axonemes). Compared to uninjected, SC-KD injection produced little to no effect on axoneme length. In contrast, the kinocilia in Smc3-KD larvae exhibited a significant (approximately 41%) decrease in axoneme length (Fig. 1A,B). Thus, Smc3 appears to contribute to kinocilia growth and/or dynamics.

To validate that the observed phenotypes are not due to non-specific defects associated with morpholino injections, we attempted to rescue the kinocilia length defects by overexpressing the *smc3* mRNA. The *smc3* mRNA was co-injected in Smc3-KD or SC-KD zygotes at the one cell stage. Embryos were raised to 3 dpf, fixed, and processed to provide for kinocilium-containing neuromast detection along the posterior lateral line. Axoneme length was evaluated as described above. Co-injection of *smc3* mRNA in Smc3-KD embryos produced significantly longer kinocilia than in Smc3-KD larvae alone, demonstrating that exogenous *smc3* mRNA can partially rescue kinocilia length (Fig. 1C,D). Interestingly, SC-KD larvae co-injected with *smc3* mRNA also displayed shorter kinocilia than SC-KD larvae alone, suggesting that normal kinocilium length depends on a tightly regulated level of Smc3. Next, to rule out the possibility that the kinocilia length phenotype is due all or in part to a developmental delay, we compared the number of somites between Smc3-KD and SC-KD larvae at 3 dpf. A range of 27 to 31 somites were visible across larvae of each injection type, and there was no significant difference between the number of somites present between Smc3-KD and SC-KD (Fig. S3). Together, the findings that the *smc3* mRNA rescued the kinocilia length defects, that *smc3* overexpression similarly causes kinocilia length defects (i.e. suggesting that Smc3 protein levels are the key variable impacting kinocilia length), that SC-KD alone did not cause kinocilia defects, and that there is not a generalized developmental delay, indicate that the observed Smc3-KD phenotypes are not due to non-specific defects associated with morpholino injections.

**Fig. 2.**
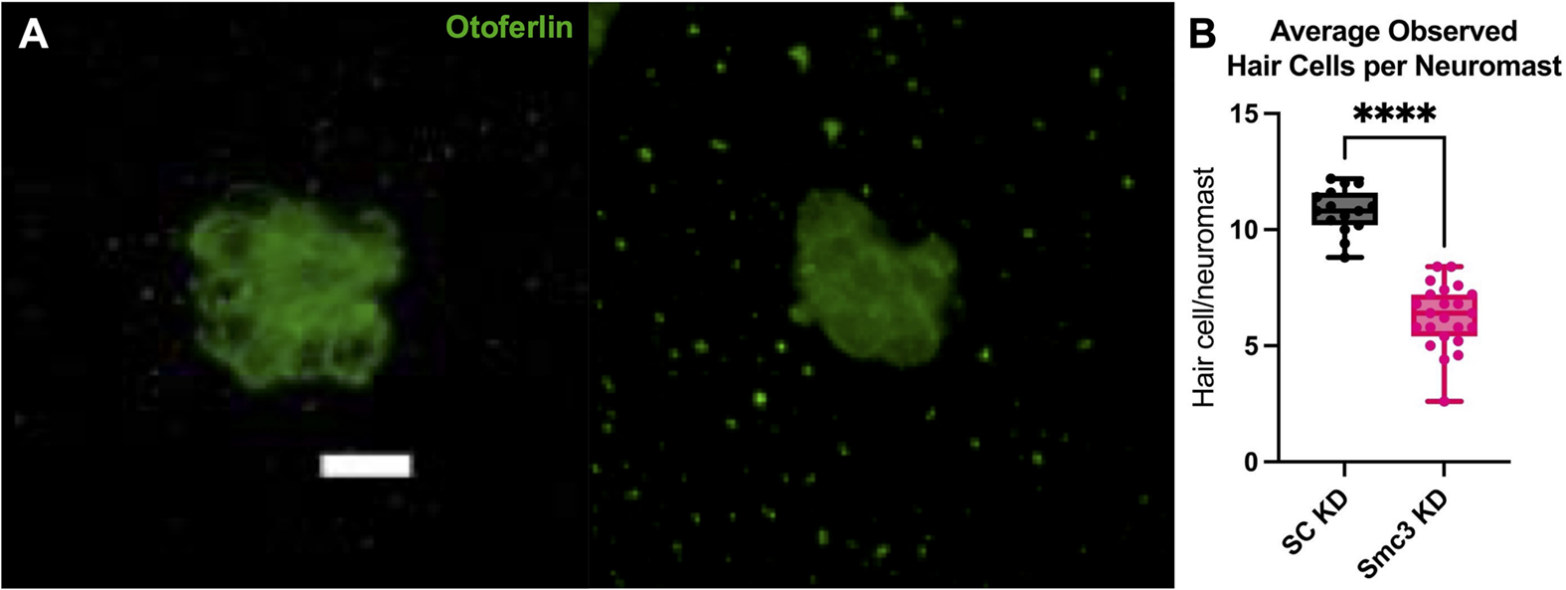
**Smc3 KD results in decreased hair cell number in neuromasts along the lateral line.** (A) 3D maximum projection representative images of SC-KD (left) and Smc3-KD (right) MI1 neuromasts observed. Hair cells are visualized with an HCS-1 antibody at a 1:100 dilution to label Otoferlin. Scale bar: 8.4 µm in the x and y dimensions. (B) Quantification of the decrease in observed hair cells per neuromast in 5 dpf Smc3-KD larvae as compared to SC-KD. Student's *t*-test, *P*<0.0001. Five neuromasts were observed per embryo *n*=15, Smc3-KD embryo *n*=23.

To further evaluate the influence of Smc3 on hair cells, we quantified hair cell number in select neuromasts along the lateral line at 5 dpf (note that Smc3 protein levels are reduced by about 71% at 5 dpf in Smc3-KD embryos, Fig. S1). Compared to SC-KD, Smc3-KD embryos exhibited about 42% fewer hair cells per neuromast (Fig. 2). This indicates that in addition to influencing kinocilia length, reduced Smc3 expression also impacts either hair cell proliferation, differentiation, or survival.

### Smc3 contributes to kinocilia function

**Fig. 3.**
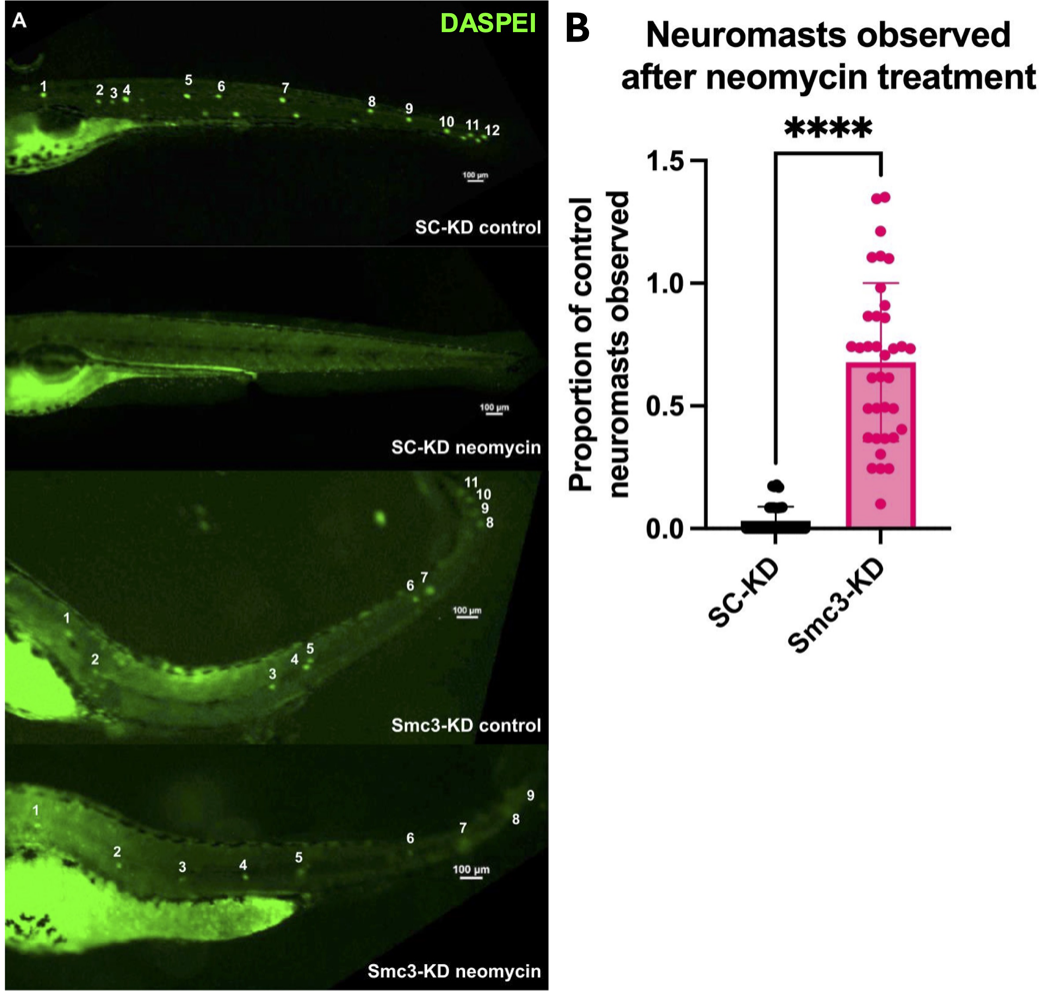
**Smc3 protein depletion protects lateral line neuromasts from neomycin cytotoxicity.** (A) Representative images of lateral line neuromasts in SC-KD larvae and Smc3-KD larvae at 5 dpf with and without neomycin treatment and stained with 2-[4-(Dimethylamino)styryl]-1-ethylpyridinium iodide (DASPEI). Scale bars: 100 µm. (B) Quantification of neuromast survival after neomycin treatment for each injection type. Neuromast survival was calculated as the ratio of observed neuromasts in each neomycin-treated embryo to the average number of neuromasts visible in control-treated embryos of the same injection type (e.g. SC-KD or Smc3-KD). By this method, ratios greater than 1 may occur if the number of observed neuromasts in a neomycin-treated embryo exceeds the average number of neuromasts observed in the relevant control-treated embryos. Student's *t*-test, ****P<0.0001, SC-KD *n*=40, Smc3-KD *n*=36.

Given the adverse impact of Smc3-KD on kinocilia length and hair cell number, it became important to test the extent to which Smc3-KD also impacted kinociliary function. Neomycin is an aminoglycoside antibiotic that at high doses produces mechanosensory hair cell loss in the human ear and in zebrafish neuromasts (Selimoglu, 2007; Malicki et al., 2011; Harris et al., 2003). In contrast, zebrafish with mutations in cilia genes often exhibit resistance to aminoglycoside cytotoxicity (Owens et al., 2008; Stawicki et al., 2016; Wang et al., 2022). To test whether the reduced kinocilia length that occurs in response to Smc3-KD also influences neomycin sensitivity, neuromast survival was monitored in larvae treated for Smc3-KD. Embryos were injected with either SC-MO or the *smc3*-MO, raised to 5 dpf, and then treated with neomycin. Viable neuromasts were detected using the cationic styrenyl dye 2-[4-(Dimethylamino)styryl]-1-ethylpyridinium iodide (DASPEI). This voltage-sensitive dye is quickly taken up by sensory hair cells along the lateral line, and therefore specifically labels neuromasts following exposure (Harris et al., 2003). As expected, neomycin treatment in SC-KD embryos caused death of the hair cells along the lateral line. This was demonstrated by loss of DASPEI staining as compared to SC-KD embryos not exposed to neomycin, where all 12 neuromasts were readily detectable (Fig. 3). In contrast, Smc3-KD larvae appeared to be protected against neomycin-induced hair cell death. We note that Smc3-KD alone resulted in fewer neuromasts in the posterior line, and these neuromasts were not evenly spaced (however, whether altered number or deposition of neuromasts is a direct or indirect outcome of Smc3-KD is unknown). Even so, treatment with neomycin resulted in less hair cell death than in SC-KD (Fig. 3). When presented as a ratio of the number of neuromasts present after neomycin treatment, compared to the number of neuromasts present without neomycin treatment, we find that SC-KD larvae showed a 96% loss of neuromasts with neomycin treatment, while only about a third of neuromasts were lost with neomycin treatment in Smc3-KD larvae. In combination, these results suggest that Smc3-KD results in dysfunctional kinocilia.

**Fig. 4.**
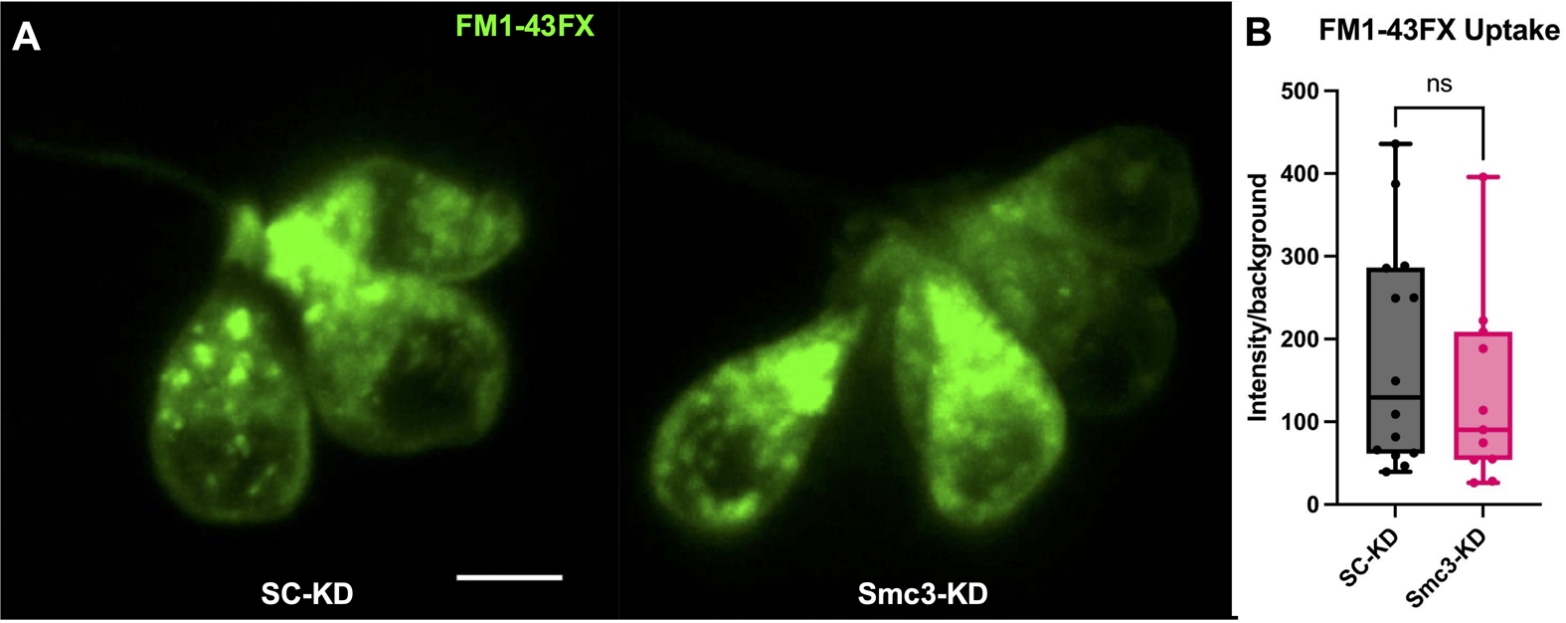
**Smc3 protein depletion does not significantly impact FM1-43FX uptake via mechanotransduction.** (A) Maximum projection representative images of first posterior lateral line neuromasts of each injection type following brief FM1-43FX treatment in 6 dpf embryos. (B) Quantification of the average fluorescence intensity normalized to background fluorescence of neuromasts of each injection type. Scale bar: 10.4 µm in the x and y dimensions. Student’s *t*-test, *P*=0.3646, SC-KD *n*=14, Smc3-KD *n*=11.

The mechanism of neomycin resistance in cilia mutants is often due to reduced neomycin loading (Hailey et al., 2012; Stawicki et al., 2014; Stawicki et al., 2016). Neomycin loading into hair cells requires mechanotransduction channels (Gale et al., 2001; Marcotti et al., 2005; Alharazneh et al., 2011). Therefore, we next investigated the uptake of FM1-43X, a vital dye that also requires mechanotransduction activity for cell entry (Meyers et al., 2003). As expected, SC-KD larvae exhibited robust FM1-43X uptake (Fig. 4). Interestingly, Smc3-KD larvae also exhibited strong uptake of FM1-43X. These results are similar to the IFT mutant *wdr35*, and to several transition zone mutants, which exhibit both neomycin resistance and normal FM1-43X uptake (Stawicki et al., 2016). Notably, this same group of mutants exhibit only modest defects in neomycin loading. Overall, these studies suggest that neomycin and FM1-43X enter cells via different (although possibly overlapping, Patterson et al., 2024) mechanosensitive pathways.

## DISCUSSION

The present study provides the first functional data showing that Smc3 contributes to hair cell activities. First, we found that Smc3-KD and *smc3* mRNA overexpression each cause reduced kinocilia length. This indicates that Smc3 protein levels are tightly coordinated with kinocilia assembly, disassembly, and/or dynamics. Next, we found that Smc3-KD influences hair cell number, suggesting that Smc3 contributes to cell proliferation, differentiation, or survival. We further showed that Smc3-KD promotes neomycin resistance but does not disrupt FM1-43X loading. We favor a model where Smc3 functions autonomously in hair cells to cause kinocilia length and hair cells number defects. However, because of the systemic nature of the Smc3-KD and the ubiquitous nature of Smc3 function, we cannot rule out the possibility that the observed defects are due to non-autonomous effects. For example, it is not clear if Smc3 function in the neuromast support cells could cause these phenotypes. Whether acting autonomously or not, the observed structural and functional kinocilial defects in Smc3-KD larvae are interesting given the influence of both cohesins and cilia on the development of overlapping tissues and organs (Mill et al., 2023; Hyland and Brody, 2021; Piché et al., 2019).

Unexpectedly, hair cells in *smc3* morphants exhibit both neomycin resistance and normal uptake of the mechanotransduction-dependent dye FM1-43X, despite that loading of both neomycin and FM1-43X require mechanotransduction (Gale et al., 2001; Marcotti et al., 2005; Alharazneh et al., 2011; Meyers et al., 2003). However, there are several examples of kinocilia mutants that similarly exhibit neomycin resistance but unaffected uptake of FM1-43X (i.e. *wdr35*, *cc2d2a*, *mks1*, *cep290*; Stawicki et al., 2016) Thus, there may be multiple mechanisms mediating mechanotransduction and/or neomycin resistance in hair cells. For example, we cannot rule out the possibility that neomycin resistance in the *smc3* morphants occurs via a kinocilia-independent mechanism.

Future studies will be aimed at revealing the underlying mechanism for how Smc3 impacts kinocilia length and function. For example, the extent to which the canonical roles of Smc3 (i.e. chromosome cohesion and regulation of gene transcription) contribute to kinocilia length and function is unclear. Others have attributed severe Smc3 developmental phenotypes to chromosome loss/genomic instability in response to elevated doses of the *smc3*-MO (Ghiselli, 2006). Alternatively, using a lower dose of the *smc3*-MO, we have attributed less severe Smc3 phenotypes (reduced body length, smaller eyes, otolith defects, and now kinocilia length/function defects) to altered regulation of gene transcription (Sanchez et al., 2022). Yet another unexplored possibility is that zebrafish Smc3 may influence kinocilia length via a non-canonical role, such as by localizing to the basal body or axoneme (i.e. as was found for mammalian SMC3 and SMC1, Khanna et al., 2005; Guan et al., 2008). Despite these uncertainties, in combination with previous studies demonstrating cohesin protein localization to centrosomes, basal bodies, and cilia membranes (Khanna et al., 2005; Guan et al., 2008; Yang et al., 2019; Scheuerle et al., 2023; Reed et al., 2023), we propose that the findings presented here add to an emerging body of knowledge functionally linking cohesins and cilia. Continued research into these questions may therefore reveal overlapping etiologies for cohesinopathies and ciliopathies.

## MATERIALS AND METHODS

### Zebrafish maintenance

C32 zebrafish (*Danio rerio*) were used in this study. All protocols employed in this study were approved by the Institutional Animal Care and Use Committee (IACUC) at Lehigh University (Protocol 187). This study was conducted in line with recommendations put forth by the Guide for the Care and Use of Laboratory Animals of the National Institutes of Health. Lehigh University's Animal Welfare Assurance Number is A-3877-01.

### MO injections

MOs were purchased from GeneTools, LLC (Philomath, OR, USA). MOs were dissolved in dH_2_O to create a 0.5 mM concentration and heated at 65°C for 15 min prior to injection. Zebrafish were bred via *in-vitro* fertilization and zygotes were microinjected with either SC-MO (CCTCTTACCTCAGTTACAATTTATA) or protein-depleting MO (*smc3*-MO1: TGTACATGGCGGTTTATGC; Banerji et al., 2017) at the 1-cell stage using the Narishige IM 300 Microinjector (about 1-2 nl injection volume). Embryos were sorted for viability and fertilization and raised to indicated age at 28°C in egg media and ampicillin solution. All *smc3* MO and corresponding SC-MO microinjections had concentrations of 0.5 mM and corresponded to about 3-5 ng of MO. Before all experiments, embryos were de-chorionated as needed with pronase. For immunofluorescence experiments, embryos were then fixed in 4% paraformaldehyde (PFA) overnight at 4°C or for 4 h at room temperature, washed with 1X PBS, and stored in 1X PBS at 4°C before immunofluorescence protocol.

### mRNA injection

Full length mRNA encoding for *smc3* was designed using sequences from the Zebrafish Information Network (ZFIN) database and inserted in a pcDNA3.1(+) plasmid backbone. The sequence for *smc3* mRNA was sequenced to confirm correspondence with NCBI (NM_214689). Prior to transcription reactions using the Invitrogen mMessage mMachine kit, the plasmid was linearized by performing a NotI digest. The concentration of the resulting mRNA was assessed using the Thermo Scientific Nanodrop 2000 and mRNA integrity was assessed on a formaldehyde gel and imaged using the Bio-Rad Gel Doc. The mRNA was then diluted to a concentration of 100 ng/μl in Phenol Red and RNAse free water. Diluted mRNA was heated at 65°C for 5 min prior to injections into zebrafish embryos at the 1-cell stage as previously described (about 1-2 nl injection volume). All embryos were fixed at 72 h post fertilization in 4% PFA overnight at 4°C or for 4 h at room temperature for further immunofluorescent staining.

### Imaging analysis

For live imaging, zebrafish larvae were anesthetized in 0.016% tricaine and mounted on double cavity slides. 3% methyl cellulose was used for embedding, though care was taken to not push embryos into the methyl cellulose to avoid suffocation. Larvae were observed and imaged at room temperature using a Nikon Eclipse 80i Microscope and SPOT-RTKE digital camera (Diagnostic Instruments) and SPOT software (Diagnostic Instruments) that the microscope is equipped with. Embryos were positioned laterally and imaged at 4X magnification.

### Kinociliary length imaging

Zebrafish larvae were fixed at 3 dpf and stored in glass vials 1X PBS at 4°C. Embryos were permeabilized with 2% Triton X-100 for 3 h at room temperature with agitation. Larvae were washed four times for 5 min each with 1X PBS at room temperature, then blocked for 1 h at room temperature on a shaker using a blocking solution containing 1% bovine serum albumin (ThermoFisher, BP1600-100), 0.1% DMSO (Sigma-Aldrich, D2650), and 5% normal goat serum (ThermoFisher, 10000C). The BDP block with 5% normal goat serum was prepared by mixing 0.1 g bovine serum albumin with 10 μl dimethyl sulfoxide and 500 μl normal goat serum in a 15 ml centrifuge tube. 1X PBS was then added to the vial until the solution reached the 10 ml line on the centrifuge tube and the solution was mixed until homogeneous. After blocking, larvae were washed four times for 5 min each with 1X PBS at room temperature and then incubated in primary antibodies overnight at 4°C with agitation. The primary antibody used was a 1:300 dilution of acetylated tubulin (Sigma-Aldrich, T6793). Larvae were then washed four times for 5 min each with 1X PBS at room temperature and then incubated in secondary antibodies and DAPI for 2 h at room temperature on a shaker. The secondary antibodies used were a 1:1000 donkey anti-mouse 647 (Invitrogen, A31571) to label acetylated tubulin and a 1:1000 dilution of DAPI. Vials were wrapped in tinfoil at this and all future steps to prevent photobleaching. Larvae were washed five times for 5 min each with 1X PBS at room temperature and stored in 1X PBS at 4°C until imaging.

### Confocal microscopy

Immunofluorescent-stained larvae were embedded in a 50% glycerol 50% 1X PBS solution between two 22mmX60mm microscope coverslips separated by a stack of two 22mmX22mm microscope coverslips secured to one of the larger coverslips with clear superglue. Larvae were positioned laterally and then observed and imaged on a Zeiss LSM800 scanning confocal microscope. Larvae were observed and imaged using the 40X objective combined with a 4X magnification. A stack of images was captured to image the first neuromast along the posterior lateral line. The number of optical sections varied to ensure that the entire neuromast was captured in the stack. The depth of optical sections varied from 1.16-1.17 μm and was set based on the optimal section depth indicated by Zen software. All optical section stacks were saved as CZI files.

### Somite counts

Larvae at 3 dpf Smc3-KD and SC-KD were mounted laterally in 3% methyl cellulose and imaged as outlined in the ‘Image analysis’ section. Multiple images of each individual were captured to ensure that the entire length could be visualized in focus for accurate counting. Somites were identified and counted based on Stickney et al. (2000). Somite counts of Smc3-KD and SC-KD larvae were compared using an unpaired Student’s *t*-test.

### Hair cell immunolabelling and counts

Protocol was adapted from Galdieri et al. (2023). Zebrafish larvae were fixed at 5 dpf and stored in glass vials 1X PBS at 4°C and immunostained using the same protocol as done for staining to visualize axonemal length. To visualize hair cells, larvae were stained with mouse anti-otoferlin (Developmental Studies Hybridoma Bank, HCS-1) at a 1:100 dilution. The secondary antibodies used was donkey anti-mouse 647 (Invitrogen, A31571) at a 1:700 or 1:800 dilution. Larvae were observed and imaged on a Zeiss LSM800 scanning confocal microscope using the 40X objective. The number of hair cells in neuromasts MI1, MI2, O2, MI2, and IO4 (Raible and Kruse, 2000) were counted and recorded. Each larva's total number of hair cells in these neuromasts was divided by 5 to obtain an average number of hair cells per neuromast.

### Axoneme length analysis

CZI files were imported into Fiji and the 647 channel (which was used to detect acetylated tubulin) was used to evaluate kinociliary length. The x and y coordinates and the optical slice (z-slice) number were recorded for the tip and base of the kinocilium. The x- and y-displacements were calculated by finding the difference between the x and y coordinates, respectively, at the axoneme tip and base. The 2D displacement was estimated as the hypotenuse of a right triangle formed with the x-displacement and y-displacement as the legs.

The z-displacement was calculated as the number of z-slices between the axoneme tip and base multiplied by the width of each z-slice. The axoneme length was then estimated as the hypotenuse of a right triangle formed with the 2D displacement and the z-displacement as the legs. Axoneme lengths were compared between treatment and control groups using the Student's *t*-test.

### Neomycin treatment, neuromast staining, and analysis

Protocol adapted from Stawicki et al. (2016). 5 dpf larvae were placed in 60×15 mm Petri dishes with either 50 ml of 200 μM neomycin in sterile embryo media (EM) or 50 ml of sterile EM for 30 min at 28°C. Larvae were washed three times for 5 min each in sterile EM at 28°C, then allowed to recover for 1 h in sterile EM at 28°C. Embryos were incubated in a 75 μg/ml dilution of 2-[4-(dimethylamino) styryl]-N-ethylpyridinium iodide (DASPEI) at room temperature for 30 min to label neuromasts and were imaged at 4X magnification on a Nikon Eclipse 80i Microscope equipped with a SPOT-RTKE digital camera and SPOT software using X-Cite 120 Fluorescence Imaging System as a fluorescent source. Multiple images of each were captured at different focuses to ensure visualization of all stained neuromasts if not all were in focus on the same plane. Saturation and brightness increased on representative images to assist in neuromast visualization in Fig. 3.

Image files were imported into Fiji and the number of visible neuromasts were recorded for each embryo. The ratio of neuromast survival was calculated by dividing the number of neuromasts visible in a neomycin-treated embryo by the average number of neuromasts visible on control-treated embryos of the same injection type (e.g. SC-KD or Smc3-KD). By this method, ratios greater than 1 may occur if the number of observed neuromasts in a neomycin-treated larvae exceeds the average number of neuromasts observed in the relevant control-treated larvae. SC-KD and Smc3-KD ratios of neuromast survival were compared using a Student's *t*-test.

### FM1-43X Staining and analysis

Protocol adapted from Parkinson and Stawicki (2021). Larvae at 6 dpf were treated 2.25 μM FM 1-43FX (ThermoFisher, F35355) for 1 min at room temperature, then promptly removed, washed 3 times for 5 min each in sterile egg media, and anesthetized with 0.4% tricaine. Larvae were imaged using a Zeiss LSM800 scanning confocal microscope. The first posterior lateral line neuromast was imaged from the cell body to the axonemal tip by capturing a stack of 1 um sections. The average fluorescent intensity of neuromast cell bodies was measured in Fiji using a maximum projection image of each stack. This measurement was normalized to the background by measuring the average fluorescent intensity of a large region of the background of each image. Average fluorescent intensities of Smc3-KD and SC-KD larvae were compared using the Student's *t*-test. Outlier data points falling above or below the interquartile range of the data set were excluded from analysis and data visualization in Fig. 4B (i.e. 9 SC-KD and 15 Smc3-KD data points were excluded). Exclusion of outliers did not change the results of analysis.

### Protein lysates and immunoblotting

SC-KD and Smc3-KD lysates were prepared at 3 dpf and 5 dpf using a protocol adapted from Schnabel et al. (2019). Larvae were de-chorionated as needed with pronase and washed with E3 egg water. About 10-12 larvae were placed in 1.5 µl centrifuge tubes and all excess egg water was removed from the tubes. Next, 500 ml of cold heptane was added, immediately followed by 500 µl of cold methanol to the tubes and samples were fixed for 5 min. Samples were washed twice with 500 µl of cold methanol, then twice with 100 µl of 1X RIPA buffer. Larvae were homogenized in 10 µl of 1X RIPA buffer per larva, then mixed with the same amount of 4X sample buffer. Lysates were then heated at 95°C for 5 min and stored at −80°C.

Prior to use, samples were boiled for 5 min at 95°C. 20 µl of sample was loaded and electrophoresed on 10% sodium dodecyl-sulfate polyacrylamide gel electrophoresis gels. Protein expression was evaluated via fluorescent western blotting as described in Farwell et al. (2016). Blots were blocked and incubated overnight at 4°C in rabbit anti-Smc3 (1:200, Abcam ab9263) and mouse anti*-α*-Tubulin (1:200, ThermoFisher T6074) in 2% BSA/Tris-buffered saline with Tween-20 (TBST). The following day, Alexa 488 anti-rabbit (1:500 Invitrogen A11008) and Alexa 647 anti-mouse (1:500, Invitrogen A31571) were diluted in TBST and incubated with the blots for 2 h. Imaging was performed using the ChemiDoc MP Imaging System (Bio-Rad 170-8280). Band intensity analysis was performed using ImageJ (NIH). Relative pixel intensities of each band were normalized to local background intensity. *α*-Tubulin was used as a loading control, and therefore Smc3 band intensities were normalized to the corresponding *α*-Tubulin band intensity. Protein depletion was analyzed using a two-tailed Student's *t*-test.

## Supplementary Material

10.1242/biolopen.062029_sup1 Supplementary information
