# Supplementary material for: Cohesin protein Smc3 influences kinocilial structure and function
Source: Biol Open. 2025 Dec 11;14(12):bio062029. doi: 10.1242/bio.062029 (PMC12746713; doi:10.1242/bio.062029)
Supplement: Supplementary information [file biolopen-14-062029-s1.pdf]

Figure S1

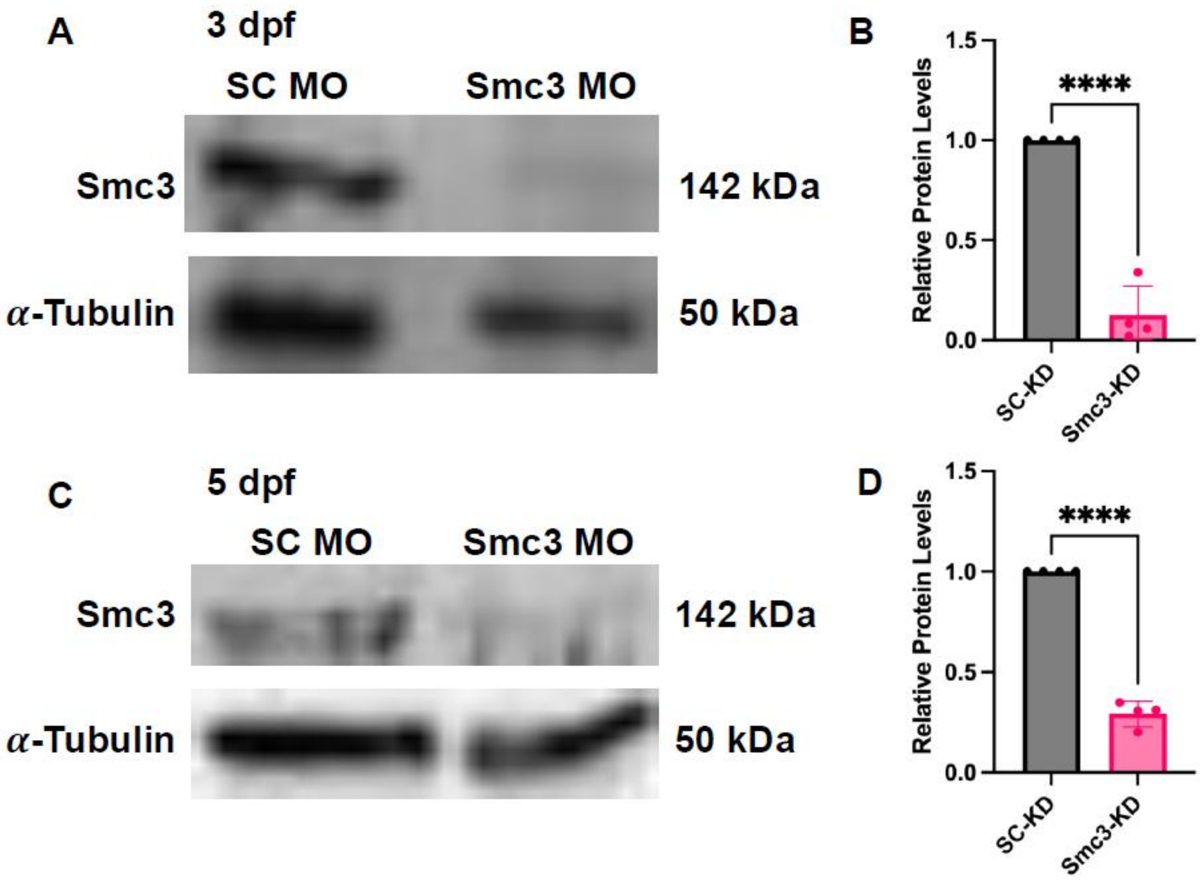

Fig. S1. Smc3 MO injections result in decreased Smc3 protein expression. (A) Western blot of SC MO 3 dpf embryo lysates compared to Smc3 MO 3 dpf embryo lysates. Immunoblots were probed with Smc3 and α-Tubulin antibodies, and each protein was detected at their predicted sizes of 142 kDa and 50 kDA, respectively. (B) Quantification of relative protein levels in Smc3-KD 3 dpf embryo lysates compared to SC-KD 3 dpf embryo lysates. Smc3 protein expression is significantly reduced by 87.4% in Smc3 MO injected embryos compared to SC MO injected embryos at 3 dpf (Student's t-test, $P<0.0001$, SC MO n= 4, Smc3 MO n=4). (C) Western blot of SC MO 5 dpf embryo lysates compared to Smc3 MO 5 dpf embryo lysates. Immunoblots were probed with Smc3 and α-Tubulin antibodies. (D) Quantification of relative protein levels in Smc3-KD 5 dpf embryo lysates compared to SC-KD 5 dpf embryo lysates. Smc3 protein expression is significantly reduced by 70.8% in Smc3 MO injected embryos compared to SC MO injected embryos at 5 dpf (Student's t-test, $P<0.0001$, SC MO n= 4, Smc3 MO n=4).

Biology Open • Supplementary information

Figure S2

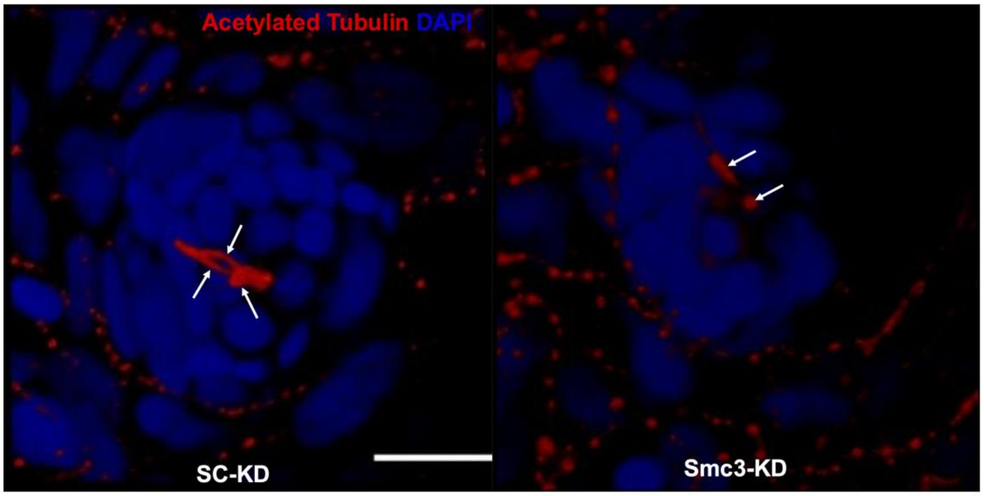

Fig. S2. Additional 3D rendering images of first posterior lateral line neuromasts in 3dpf SC-KD (left) and Smc3-KD (right) embryos exhibiting the presence of multiple kinocilia per neuromast. Multiple kinocilia per neuromast were regularly observed in all imaged neuromasts and are easily visualized by traversing image z-stacks. Acetylated tubulin is labeled in red and DAPI in blue. Scale bar= 10.4 µm in the x and y dimensions.

Figure S3

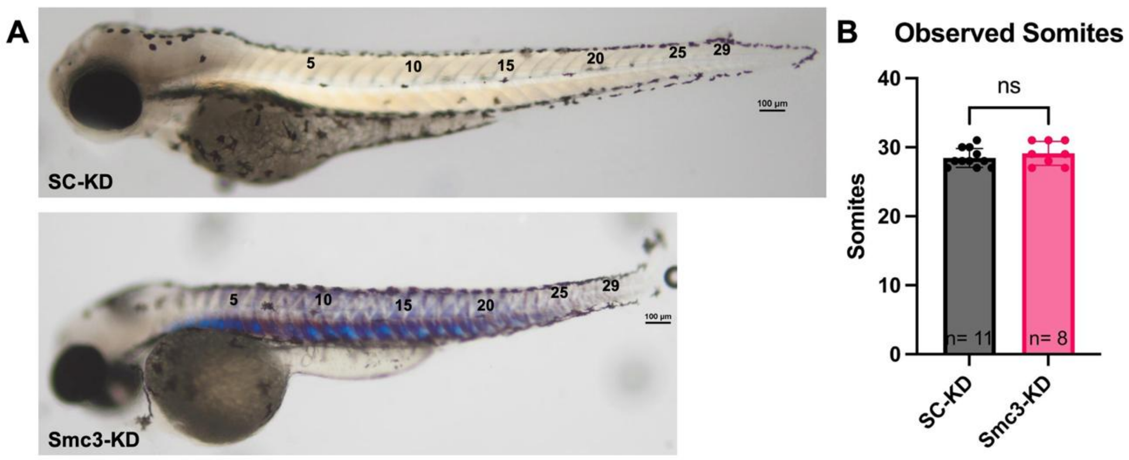

Fig. S3. Smc3-KD does not cause general developmental delay. (A) Representative images of an SC-KD (top) and Smc3-KD (bottom) 3 dpf embryo fixed in 4% PFA at 3 dpf. Every fifth somite and the last somite counted are labeled. Multiple images of each embryo were taken to ensure all somites were in focus in at least one of the images, and somites were counted using all images of a single embryo. Scale bar = 100 µm. (B) Quantification of the difference in observed somites between the SC-KD and Smc3-KD injection groups. No significant difference was found. Student's t-test, P=0.3577, SC-KD n=11, Smc3-KD n=8.
